# PI(4,5)P2 controls slit diaphragm formation and endocytosis in *Drosophila* nephrocytes

**DOI:** 10.1007/s00018-022-04273-7

**Published:** 2022-04-18

**Authors:** Maximilian M. Gass, Sarah Borkowsky, Marie-Luise Lotz, Rebecca Siwek, Rita Schröter, Pavel Nedvetsky, Stefan Luschnig, Astrid Rohlmann, Markus Missler, Michael P. Krahn

**Affiliations:** 1 Medical Cell Biology, Medical Clinic D, University Hospital of Münster, Albert-Schweitzer Campus 1-A14, 48149 Munster, Germany; 2 Institute of Integrative Cell Biology and Physiology, University of Münster, Schlossplatz 8, 48143 Munster, Germany; 3 Institute of Anatomy and Molecular Neurobiology, University of Münster, Vesaliusweg 2-4, 48149 Munster, Germany

**Keywords:** Nephrocyte, Podocyte, Slit diaphragm, Phosphoinositides, PI3-kinase, Phospholipids, PTEN

## Abstract

*Drosophila* nephrocytes are an emerging model system for mammalian podocytes and proximal tubules as well as for the investigation of kidney diseases. Like podocytes, nephrocytes exhibit characteristics of epithelial cells, but the role of phospholipids in polarization of these cells is yet unclear. In epithelia, phosphatidylinositol(4,5)bisphosphate (PI(4,5)P2) and phosphatidylinositol(3,4,5)-trisphosphate (PI(3,4,5)P3) are asymmetrically distributed in the plasma membrane and determine apical–basal polarity. Here, we demonstrate that both phospholipids are present in the plasma membrane of nephrocytes, but only PI(4,5)P2 accumulates at slit diaphragms. Knockdown of Skittles, a phosphatidylinositol(4)phosphate 5-kinase, which produces PI(4,5)P2, abolished slit diaphragm formation and led to strongly reduced endocytosis. Notably, reduction in PI(3,4,5)P3 by overexpression of PTEN or expression of a dominant-negative phosphatidylinositol-3-kinase did not affect nephrocyte function, whereas enhanced formation of PI(3,4,5)P3 by constitutively active phosphatidylinositol-3-kinase resulted in strong slit diaphragm and endocytosis defects by ectopic activation of the Akt/mTOR pathway. Thus, PI(4,5)P2 but not PI(3,4,5)P3 is essential for slit diaphragm formation and nephrocyte function. However, PI(3,4,5)P3 has to be tightly controlled to ensure nephrocyte development.

**Supplementary Information:**

The online version contains supplementary material available at 10.1007/s00018-022-04273-7.

## Introduction

In *Drosophila*, pericardial nephrocytes located along the heart tube and garland nephrocytes surrounding the proventriculus filtrate the hemolymph and endocytose proteins and toxins to store the latter permanently to inactivate them [1]. Nephrocytes were shown to share several key features with podocytes in vertebrates, qualifying them as a model system to study mammalian podocyte function and podocyte-associated diseases [2–4]. Like in podocytes, homologues of Nephrin- and Neph1 (Sticks and stones (Sns)/Hibris (Hbs) and Kin of irre (Kirre)/Roughest (Rst)) form the slit diaphragm, thereby separating the lacunae from the body cavity with hemolymph. These lacunae are formed by invaginations of the plasma membrane and form channel-like structures with both ends connected to the extracellular space [5]. Due to the high endocytosis capacity in these lacunae and the expression of endocytosis receptors like Cubilin, Megalin and Amnionless, nephrocytes are used as a model system for proximal tubules of the kidney, too [6].

Apart from the core components of the Nephrin/Neph1 family, the slit diaphragm is stabilized by adapter proteins, e.g. the Podocin homologue Mec2 [1, 7] and the ZO-1 homologue Polychaetoid [1, 8]. Furthermore, we recently showed, that regulators of classical apical–basal polarity in epithelia are partly localized to slit diaphragm complexes [9]. Knockdown studies revealed that apical polarity regulators, such as Crumbs/Stardust and the PAR/aPKC complex as well as the basolateral polarity determinants Scribble/Lethal (2) giant larvae and PAR-1 are essential for slit diaphragm formation and—at least some of them—for endocytosis [9–11].

In classical epithelia, these polarity regulators are targeted to either the apical (Crumbs- and PAR/aPKC-complex) or the basolateral (Scribble/Dlg/Lgl-complex, PAR-1/LKB1) plasma membrane and are essential for the establishment and maintenance of apical–basal polarity and cell–cell contacts [12]. However, not only proteins are involved in this process, but also distinct phospholipids are enriched either in the apical or the basolateral plasma membrane: in particular, phosphatidylinositol(4,5)bisphosphate (PI(4,5)P2) accumulates in the apical membrane, whereas phosphatidylinositol(3,4,5)trisphosphate (PI(3,4,5)P3) is preferentially found in the basolateral membrane domain [13, 14]. Notably, PTEN, which dephosphorylates PI(3,4,5)P3 to generate PI(4,5)P2, is recruited to the plasma membrane by PAR-3, the core scaffolding protein of the PAR/aPKC complex [15–17]. Thereby, junctionally localized PAR-3/PTEN establishes a segregation point for PI(3,4,5)P3 and PI(4,5)P2 [13]. In turn, PAR-3 directly binds to PI(4,5)P2 and PI(3,4,5)P3, which contributes to its targeting to the plasma membrane [18, 19]. During epithelial polarization, phosphatidylinositol-3-kinase (PI3K), which phosphorylates PI(4,5)P2 to PI(3,4,5)P3, seems to function as one of the first cues to determine the basolateral, PI(3,4,5)P3-enriched plasma membrane domain [20]. Moreover, disruption of the PI(4,5)P2/PI(3,4,5)P3 balance results in severe polarity defects, suggesting a role of phospholipids as determinants of apical–basal cell polarity [13, 14].

Apart from their function in polarity, PI(4,5)P2 and PI(3,4,5)P3 are involved in various cellular processes, e.g. both phospholipids are essential regulators of Actin cytoskeleton [21–23], PI(,5)P2 enriched microdomains of the plasmamembrane are docking points for both exo- and endocytotic vesicles [reviewed by 24] and PI(3,4,5)P3 activates the Phosphoinositide-dependent kinase 1 (PDK1)–Akt pathway, thereby enhancing mTOR activity, which functions as a key regulator of cell growth, energy homeostasis and proliferation.

Although podocytes and nephrocytes share key features with classical epithelial cells, like cell–cell junctions and apical–basal polarity, little is known about the distribution and function of PI(4,5)P2 and PI(3,4,5)P3 in these cell types. Moreover, several studies suggest different functions of PI3K and PTEN in cultured podocytes [25–28], but the role of these key enzymes in vivo is still unclear. Therefore, the aim of this study was to investigate the subcellular accumulation of these two phospholipids as well as their function in slit diaphragm assembly and nephrocyte development.

## Materials and methods

### Drosophila stocks and genetics

Fly stocks were cultured on standard cornmeal agar food and maintained at 25 °C. For downregulation or overexpression of specific genes for immunostainings and electron microscopy, *sns::GAL4* [29], was crossed with the following lines: UAS::Akt-RNAi (#103703), UAS::Exo70-RNAi (#103717), UAS::Or83b-RNAi (negative control, #100825), UAS::PTEN-RNAi (#01475), UAS::Sec3-RNAi (#108085), UAS::Sktl-RNAi (#101624), UAS::Sns-RNAi (#109442) (provided by Vienna *Drosophila* Resource Center, Austria), UAS::PI3K92E-CAAX (PI3K-CA, #8294), UAS::PI3K92E.A2860C (PI3K-DN, #8289), UAS:Sktl (#39675), UAS::PH(PLCδ)-mCherry (#51658), tubP::GAL80ts (65406), UAS::dTOR-RNAi (#34639) (all obtained from Bloomington stock center). UAS::Myr::Akt was provided by Hugo Stocker [30] and UAS::Myc-Sktl was obtained from Sandra Claret [31].

Dorothy(Dot)::GAL4 [32] was provided by Achim Paululat. attP40 was used as control for PI3K-CA. UASt::PTEN was established by PhiC31-Integrase-mediated germ line transformation using attP86F. UAS::PH(Akt)-GFP was constructed by fusing the PH domain of mammalian Akt1 to the N-terminus of GFP in the pUASt vector. Transgenic flies were generated by P-element-mediated germ line transformation. An insertion on second chromosome was used in this study. For all RNAi and overexpression experiments, crosses were kept for 3 days at 25 °C and larvae subsequently shifted to 29 °C, to obtain maximum expression. PH(PLCδ)-mCherry was expressed at 25 °C, PH(Akt)-GFP was analyzed at 18 °C, 21 °C and 25 °C, with best results at 18 °C, because at higher temperature, the expression of the chimeric protein was too strong and found overall the cell, likely due to the limited amount of PI(3,4,5)P3 to bind to.

### Endocytosis assays

For the ANP-2xGFP accumulation assay, garland nephrocytes from wandering third instar larvae were dissected in HL3.1 saline [33], fixed in 4% PFA in PBS for 10 min, stained with DAPI for 20 min, washed with PBS, and mounted in Mowiol. ANP-2xGFP accumulation per nephrocyte area (CTCF = Corrected Total Cell Fluorescence) was analyzed and quantified with ImageJ after subtracting the autofluorescent background of dissected larvae. For each genotype, at least 100 nephrocytes of 15 independent larvae were quantified.

For the FITC-Albumin endocytosis assay, garland nephrocytes from wandering third instar larvae were dissected in ice-cold HL3.1 saline and subsequently equilibrated for 15 min at room temperature. Nephrocytes were then incubated with 1 mg/ml FITC-Albumin (SIGMA #A9771) in HL3.1 saline for 15 min at room temperature to pulse nephrocytes. After rinsing with PBS, samples were incubated at room temperature for a 2 h chase in Schneider cell culture medium. Samples were then fixed for 10 min in 4% PFA in PBS, washed thoroughly with PBS and stained with membrane marker WGA-Alexa 555 (1:400) for 10 min. After final washing with PBS, nephrocytes were mounted in Mowiol. The endocytic uptake per nephrocyte area (CTCF) was calculated as described for ANP-2xGFP accumulation. For each genotype, at least 120 nephrocytes of at least 25 independent larvae were quantified.

### Lysates and western blot

Lysates of larvae were made with Laemmli buffer. SDS-PAGE and western blotting was performed according to standard procedures. The following primary antibodies were used: mouse anti ß-Actin (1:1000, Santa Cruz #47778), mouse anti GFP (1:500, Santa Cruz #sc-9996), rabbit anti Akt pS473 (1:2000, Cell signaling #4060), rabbit anti total Akt (1:1000, Cell signaling #4691).

### Immunohistochemistry

Garland nephrocytes were dissected as described above and heat-fixed for 20 s in boiling heat fix saline (0.03% Triton X-100). Subsequently, nephrocytes were washed three times in PBS + 0.2% Triton X-100 and blocked with 1% BSA for 1 h, incubated over night with primary antibodies in PBS + 0.2% Triton X-100 + 1% BSA, washed three times and incubated for 2 h with secondary antibodies. After three washing steps and DAPI-staining, nephrocytes were mounted with Mowiol. Primary antibodies used were as follows: Rabbit anti Baz [1:250, 34], rabbit anti Exo70 [1:500, 35], goat anti GFP (1:500, #600–101-215, Rockland), mouse anti Myc (1:100, 9E10, Developmental Studies Hybridoma Bank (DSHB)), guinea pig anti Rab5 (1:2000), rabbit anti Rab7 (1:2000) and rabbit anti-Rab11 (1:2000) were kindly provided by A. Nakamura [36]. mouse anti phospho-S6K (1:250, Cell signaling, #9206), rat anti RFP (1:1000, 5F8, Chromotek), chicken anti Sns [1:1000, 10], mouse anti Talin (1:20, E16B, DSHB). Secondary antibodies conjugated with Alexa 488, Alexa 568 and Alexa 647 (Life technologies) were used at 1:400. Images were taken on a Leica SP8 confocal microscope using lightning program and processed using ImageJ.

### Pearson correlation coefficient

The Pearson’s *R* value was determined to quantify colocalization by comparing pixel vs pixel using the FIJI/ImageJ plugin *Coloc 2 (Analyze/ Colocalization/ Coloc2)*. *R* values can reach from -1 (total anticolocalization) to 1 (total colocalization). The value 0 means no correlation. Before the colocalization analysis, a mask of the corresponding lightning image channel was created by setting the Fiji threshold *moments* with previous adjustments in brightness/contrast and additional background subtraction (10,0 rolling ball radius). For the subsequent Coloc 2 analysis a PSF of 3,0 and a Costes randomization of 100 were used and only *R* values with a Costes *p* value > 0,9 were considered.

### Transmission electron microscopy

Garland nephrocytes of third instar larvae were dissected in HL3.1 saline, high pressure frozen (EM-PACT2, Leica, Wetzlar, Germany), freeze-substituted in acetone/1% OsO4/5% H_2_O/0.25% uranyl acetate (AFS2, Leica, Wetzlar, Germany) and embedded in Epon. For transmission electron microscopy, 70 nm thick sections were cut using an ultramicrotome (Leica UC7, Wetzlar, Germany). All samples were imaged with a transmission electron microscope (ZEISS, Libra 120, Germany).

## Results

### PI(4,5)P2 but not PI(3,4,5)P3 is enriched at slit diaphragms

In classical epithelia, PI(4,5)P2 is enriched in the apical plasma membrane, whereas PI(3,4,5)P3 accumulates in the basolateral plasma membrane [13]. In contrast, nothing is known about the distribution of specific phospholipids in mammalian podocytes or *Drosophila* nephrocytes. Therefore, we first investigated the distribution of PI(4,5)P2 and PI(3,4,5)P3 in *Drosophila* garland nephrocytes by expressing fusion proteins consisting of a fluorescent protein and a Pleckstrin homology (PH) domain, which preferentially binds to PI(4,5)P2 (PH domain of PLCδ [37]) or to PI(3,4,5)P3 (PH domain of Akt1, this study).

**Fig. 1:**
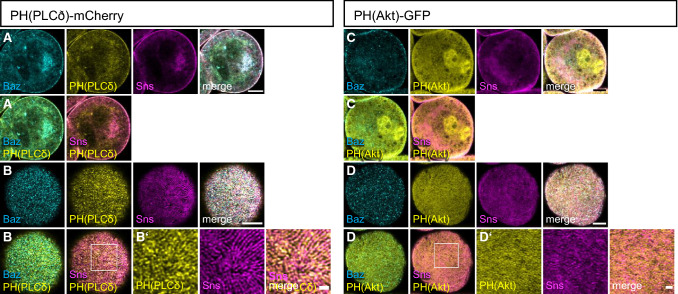
PI(4,5)P2 accumulates at slit diaphragms, whereas PI(3,4,5)P3 is rather found at the free plasma membrane. (**A**–**D**) Garland nephrocytes expressing either UAS::PH(PLCδ)-mCherry for labelling PI(4,5,)P2 or UAS::PH(Akt)-GFP to visualize PI(3,4,5)P3 were dissected from 3rd instar larvae, fixed and stained with the indicated antibodies. **A** and **C** are sections through the equatorial region of the nephrocyte; **B** and **D** are onviews onto the surface of these nephrocytes. Scale bars are 5 µm and 1 µm in insets

mCherry-PH(PLCδ) is substantially associated with the plasma membrane (Fig. 1A, B) but it is also found in intracellular pools, partly associated with vesicular structures. Surface views reveal that its cortical association form strand-like structures (Fig. 1B), which to some extent co-stain with endogenous Sns, a marker for slit diaphragms as well as with Bazooka (Baz), a key regulator of cell polarity in epithelial cells and slit diaphragm formation in nephrocytes, which directly binds to PI(4,5)P2 and PI(3,4,5)P3 [9, 19] (Supplementary Fig. 1D). In contrast, PH(Akt)-GFP is only weakly associated with the plasma membrane but rather shows a cytoplasmic and vesicular-associated distribution (Fig. 1C). Nonetheless, surface views show a strand-like pattern too, however, these strands exhibit a much weaker colocalization with Sns than mCherry-PH(PLCδ) (Fig. 1D and Supplementary Fig. 1D) and are rather found between the Sns-strands, partly colocalizing with Talin (Supplementary Fig. 1A, B). Of note, Sns and Baz pattern appear normal in nephrocytes expressing these PH domains and quantification of Sns-marked slit diaphragms revealed no differences (Supplementary Fig. 1C), confirming that expression of the PI(4,5)P2/PI(3,4,5)P3 sensors did not affect slit diaphragm formation. The functionality of these sensors was confirmed by an increased cortical accumulation upon overexpression of the PI(4,5)P2/PI(3,4,5)P3-producing enzymes (Supplementary Figs. 2I, 3D). Our findings suggest that PI(4,5)P2 in the plasma membrane accumulates at slit diaphragms, whereas PI(3,4,5)P3 is enriched in the free plasma membrane between slit diaphragms.

### Impaired PI(4,5)P2 production results in strong developmental and slit diaphragm defects

**Fig. 2:**
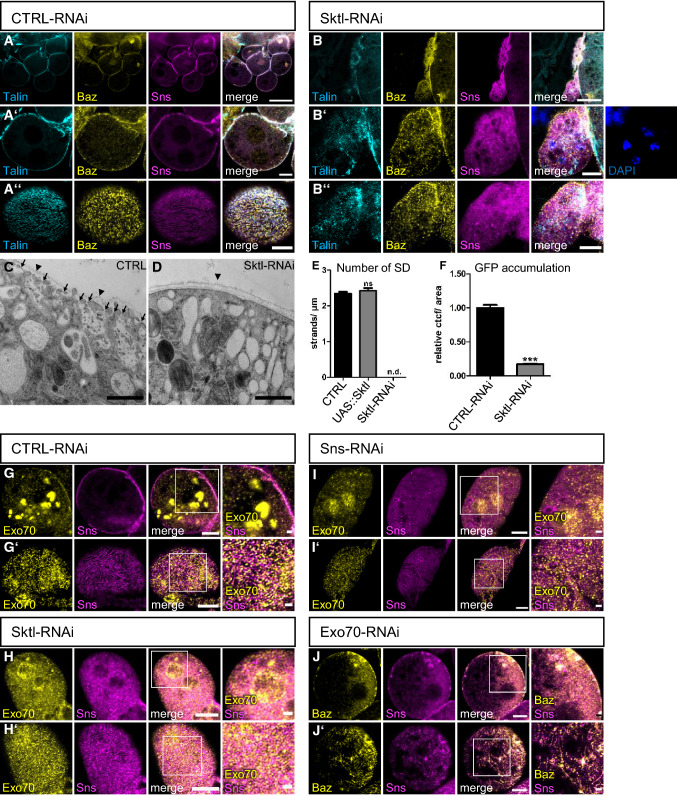
PI(4,5)P2 produced by Skittles is essential for slit diaphragm formation and endocytosis. (**A**, **B**) Garland nephrocytes from 3rd instar larvae expressing either control RNAi (**A**) or Sktl-RNAi (**B**) were stained with the indicated antibodies. (**C**, **D**) Transmission electron microscopy of garland nephrocytes of control third instar larvae (**C**) and Sktl-RNAi-expressing larvae (**D**). Some slit diaphragms were labeled with arrows in control nephrocytes. Slit diaphragms were absent in Sktl-RNAi expressing nephrocytes. Arrow heads mark the basement membrane. **E** Slit diaphragms of nephrocytes expressing Sktl or control RNAi were quantified from surface views. For this, a 5 µm line perpendicular to the Sns-strands was drawn and the number of strands quantified. 5 lines/nephrocyte and at least 5 nephrocytes were quantified per genotype. Sktl-RNAi expressing nephrocytes were not characterized as they did not display detectable Sns strands at the surface but exhibited a rather diffuse Sns staining. Significance was determined by Mann–Whitney test: n.s. not significant. n.d. not determined. **F** Endocytosis of a secreted ANP-2xGFP by garland nephrocytes expressing the indicated RNAi’s was quantified as described in the methods section. At least 100 nephrocytes from at least 15 different larvae were evaluated. Significance was determined by Mann–Whitney test: ***p < 0.001. (**G**-**I**) Nephrocytes expressing control RNAi (**G**), Sktl RNAi (**H**) and Sns RNAi (**I**) were co-stained with Exo70 and Sns. (**J**) Immunostainings of nephrocytes expressing Exo70-RNAi. Scales bars are 20 µm in A and B, 5 µm in A’, A’’, B’, B’’ and **G**–**J**, 1 µm in **C**, **D** and in insets in G’-J’. Error bars are standard error of the means

To test whether PI(4,5)P2 is essential for nephrocyte development and function, in particular regarding slit diaphragm assembly and maintenance, we used RNA interference (RNAi) to knockdown the ubiquitously expressed PI(4)P5-kinase Skittles (Sktl), which is responsible for converting PI(4)P to PI(4,5)P2 using the nephrocyte-specific driver line sns::GAL4. In *Drosophila*, Sktl has been described to regulate apical–basal polarity by targeting PAR-3 to the apical junctions in follicular epithelial cells [38] and to the anterior cortex in the oocyte [39]. In tracheal tubes, Sktl-produced PI(4,5)P2 was proposed to recruit the formin Diaphanous to the apical membrane [40]. In nephrocytes, Sktl partly colocalizes with Sns at slit diaphragms (Supplementary Fig. 2G), opening the possibility of a local accumulation of PI(4,5)P2 in microdomains of the plasma membrane at slit diaphragms. Successful reduction of PI(4,5)P2 was demonstrated by displacement of mCherry-PH(PLCδ) in nephrocytes expressing Sktl-RNAi (Supplementary Fig. 2A). Impaired expression of Sktl resulted in dramatic morphological changes with fused nephrocytes (Fig. 2B compared to control RNAi in 2A, number of nephrocytes per larvae quantified in Supplementary Fig. 3J). Furthermore, the typical strand-like structures of Sns-labelled slit diaphragm observed at the surface of control nephrocytes was completely abolished in Sktl-RNAi expressing nephrocytes, resulting in a dispersion of Sns to intracellular puncta, partly co-staining with markers for early (Rab5) and late (Rab7) endocytosis but not with Rab11 as marker for recycling endosomes (Fig. 2A–D and Supplementary Fig. 2B, C). Besides Sns, the basal polarity determinant Talin and the apical polarity regulator PAR-3 (Bazooka (Baz) in Drosophila) are lost from the cortex, too (Fig. 2B compare to A). In contrast to impaired PI(4,5)P2 levels, overexpression of Sktl in order to increase PI(4,5)P2 did not affect nephrocyte morphology or slit diaphragm assembly (Supplementary Fig. 2H, quantified in Fig. 2E), although the amount of PI(4,5)P2 seemed to be significantly increased, as demonstrated by enhanced accumulation of mCherry-PH(PLCδ) at the plasma membrane (Supplementary Fig. 2I).

### Nephrocytes with downregulation of Skittles display impaired exocytosis

Analysis of Sktl-RNAi expressing nephrocytes by electron microscopy confirmed an almost complete absence of slit diaphragms (Fig. 2D compared to control in C). Notably, these nephrocytes do not form regular lacunae but accumulate large electron-light vesicles below the plasma membrane (Fig. 2D). This phenotype suggests defects in vesicle trafficking, e.g. exocytosis, which is essential for the delivery of transmembrane proteins of the slit diaphragm complex (Sns, Kirre and Crb). During exocytosis, clustering of PI(4,5)P2 facilitates the docking of the exocyst complex to the plasma membrane by direct binding of its components Exo70 and Sec3 in yeast and in mammalian cells [41–44]. In a second step, PI(4,5)P2 is also essential for vesicle fusion and several proteins involved in regulation of fusion directly interact with PI(4,5)P2 [reviewed by 24]. To test whether Sktl-produced PI(4,5)P2 recruits exocyst complex components in nephrocytes, we stained for endogenous Exo70. In control nephrocytes, apart from intracellular giant vesicles, a substantial pool of Exo70 was found at the plasma membrane, co-localizing with Sns (Fig. 2G). In contrast, it displayed a diffuse localization with some perinuclear accumulation in Sktl-RNAi expressing nephrocytes (Fig. 2H). Moreover, downregulation of the exocyst complex components Exo70 and Sec3 resulted in similar loss of slit diaphragms as Sktl-RNAi (Fig. 2J and Supplementary Fig. 2 J), which is in line with a recent study reporting a crucial role of the exocyst complex in slit diaphragm formation/maintenance [39]. However, downregulation of Sns, resulting in impaired slit diaphragm assembly, leads to displacement of Exo70, too (Fig. 2I). These data indicate that both modules, PI(4,5)P2-enriched microdomains of the plasma membrane on the one hand and slit diaphragms on the other hand regulate Exo70 targeting and thus exocytosis.

### Decreased PI(4,5)P2 levels in nephrocytes result in decreased endocytosis

Apart from exocytosis, PI(4,5)P2 also regulates clathrin-dependent and—independent endocytosis by recruiting several proteins involved in early steps of endocytosis to the plasma membrane and by inducing actin remodeling during micropinocytosis [reviewed by 24]. In nephrocytes, endocytosis is essential for the uptake of filtrated proteins, toxins and metabolites, which are then stored and inactivated. Moreover, maintenance of the transmembrane proteins within slit diaphragms requires regulated endocytosis, too. Disturbance of slit diaphragm formation as well as of endocytic receptors and proteins involved in the endocytosis machinery have been reported to reduce endocytosis [1, 6, 10, 11, 29, 45–48]. To test, whether PI(4,5)P2 is essential for endocytosis in nephrocytes, we quantified the accumulation of secreted ANP-2xGFP [9] which is secreted into the hemolymph (quantified by Western Blot of whole third instar larvae, Supplementary Fig. 2D, E), filtrated by nephrocytes and taken up by endocytosis. Indeed, downregulation of Sktl in nephrocytes, reducing PI(4,5)P2 levels, resulted in a strong decrease of ANP-2xGFP accumulation in nephrocytes, consistent with impaired endocytosis (Fig. 2F). As the accumulation of secreted ANP-2xGFP only reflects the steady-state situation of nephrocytes in vivo and not the short-term endocytosis capacity, we performed another assay with isolated nephrocytes incubated with FITC-Albumin for 15 min and chased with normal medium for 2 h. In this short-term endocytosis assay, we found similar to the ANP-2xGFP assay that a reduction in Sktl results in strong decrease in endocytosis capacity (Supplementary Fig. 2F). Both results are in line with reports from the *Drosophila* oocyte, where Sktl is essential for Rab5-mediated endocytosis of yolk protein [49].

### Constitutively active PI3K but not enhanced PTEN or dominant-negative PI3K impairs nephrocyte morphology and function

**Fig. 3:**
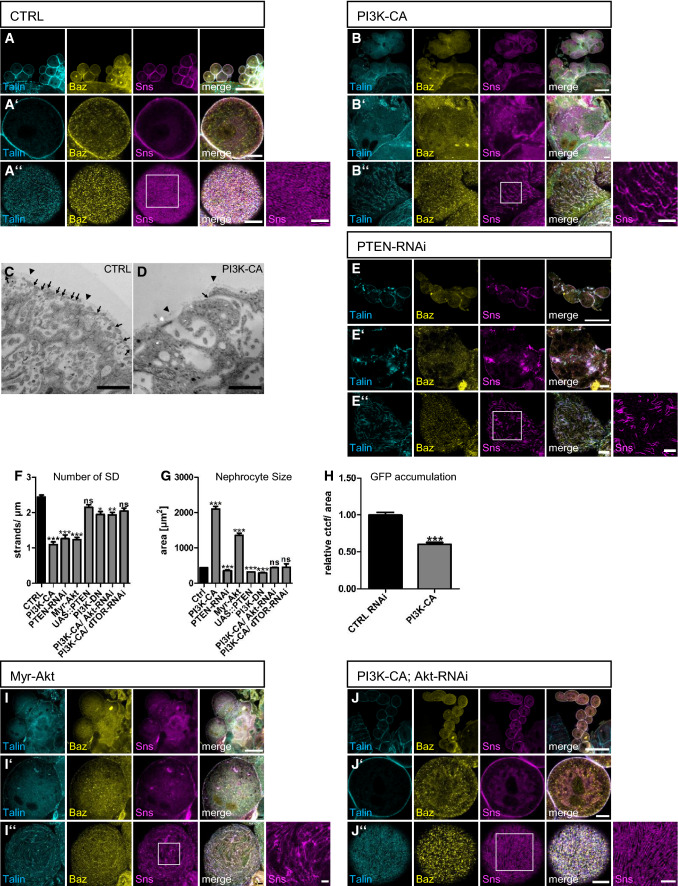
PI(3,4,5)P3 is not essential for slit diaphragm assembly and maintenance. (**A**, **B**) Garland nephrocytes from 3rd instar larvae either of controls (sns::GAL4 crossed with the empty attP40 line, (**A**) or of animals expressing a constitutively activated Pi3K (Pi3K-CA, **B**) in nephrocytes were stained with the indicated antibodies. **C**, **D** Transmission electron microscopy of garland nephrocytes of control third instar larvae (**C**) and PI3K-CA expressing larvae (**D**). Slit diaphragms were labeled with arrows and arrow heads mark the basement membrane. **E** Immunostainings of nephrocytes expressing RNAi against PTEN. **F** Slit diaphragms of nephrocytes expressing the indicated transgenes were quantified from surface views. For this, a 5 µm line perpendicular to the Sns-strands was drawn and the number of strands quantified. 5 lines/nephrocyte and at least 5 nephrocytes were quantified per genotype. Significance was determined by Kruskal–Wallis test and Dunn’s correction: ***p < 0.001, **p < 0.01, *p < 0.05, n.s. not significant. **G** The size of nephrocytes expressing the indicated transgenes was quantified by measuring the cell area of equatorial sections. At least 120 nephrocytes from at least 10 larvae were quantified. Significance was determined by Kruskal–Wallis test and Dunns correction: ***p < 0.001, n.s. not significant. **H** Endocytosis of a secreted ANP-2xGFP by garland nephrocytes expressing the indicated controls was quantified as described in the methods section. At least 100 nephrocytes from at least 15 different larvae were evaluated. Significance was determined by Mann–Whitney test: ***p < 0.001. **I** Myr-Akt expressing nephrocytes were stained with the indicated antibodies. **J** Immunostainings of nephrocytes expressing PI3K-CA together with RNAi targeting Akt. Scale bars are 50 µm in A, B, E, I and J, 5 µm in A’, A’’, B’, B’’, E’, E’’, I’, I’’, J’ and J’’, 2.5 µm in insets in A’’, B’’, E’’, I’’, J’’ and 1 µm in C and D. Error bars are standard error of the means

In contrast to PI(4,5)P2, reducing PI(3,4,5)P3 by overexpression of PTEN or expression of a dominant negative version of PI3K (PI3K-DN) did not substantially affect nephrocyte morphology or slit diaphragm formation (Supplementary Fig. 3A, B and Fig. 3F). However, overexpression of a constitutively active PI3K (PI3K-CA), which is targeted to the plasma membrane by attachment of a prenylation anchor (CAAX-motif), in nephrocytes resulted in a strong fusion phenotype and a disturbed pattern of slit diaphragms (Fig. 3A–D, quantified in 3F). Notably, PI3K-CA-expressing nephrocytes are larger than control nephrocytes and its overall number is decreased due to the fusion (Fig. 3G and Supplementary Fig. 3H, J). In addition to slit diaphragm defects, overexpression of PI3K-CA resulted in a strong decrease in ANP-2xGFP uptake and FITC-Albumin endocytosis assay, suggesting a defect in endocytosis (Fig. 3H and Supplementary Fig. 2F).

Like PI3K-CA, knockdown of PTEN, presumably resulting in enhanced accumulation of PI(3,4,5)P3, leads to similar but milder phenotypes regarding slit diaphragms, whereas cell size was not increased (Fig. 3E–G). This is likely due to the limited abundance of PI(3,4,5)P3 within the plasma membrane. Ectopic production of PI(3,4,5)P3 from PI(4,5)P2 by PI3K-CA likely produces higher levels of PI(3,4,5)P3 in the plasma membrane due to the larger pool of PI(4,5)P2 [50], whereas inhibition of dephosphorylation of PI(3,4,5)P3 to PI(4,5)P2 only moderately increases PI(3,4,5)P3 levels in the plasma membrane.

### Phenotypes of increased PI(3,4,5)P3 are induced by the Akt/mTOR pathway

Increased PI(3,4,5)P3 in the plasma membrane leads to activation of the Akt/mTOR signaling cascade, which, among various other functions, results in cell survival and increased cell size and proliferation [reviewed by 51]. Expression of PI3K-CA in nephrocytes results in a significant increase of the phosphorylation of the mTOR target S6K (pS6K, Supplementary Fig. 3E–G), conforming that increased PI(3,4,5)P production by enhanced PI3K activity results in mTOR activation. To test whether the phenotypes observed in nephrocytes expressing PI3K-CA are caused by ectopic Akt/mTOR activation, we introduced a constitutively active variant of Akt (Myr-Akt), which is recruited to the plasma membrane and activated independently of PI(3,4,5)P3 due to the fusion of a myristoylation signal [30]. Indeed, these nephrocytes mimicked the PI3K-CA overexpression phenotype with disrupted slit diaphragms, increased size and fusion phenotypes (Fig. 3F, G, I). However, cell size of Myr-Akt expressing nephrocytes was not as strongly increased as in PI3K-CA expressing ones (albeit higher than in case of PTEN-RNAi), whereas slit diaphragm assembly is severely disturbed and comparable with PI3K-CA and PTEN-RNAi-expressing nephrocytes. Thus, these data provide additional support to the notion that slit diaphragm assembly and size regulation show different susceptibility to levels of PI(3,4,5)P3.

To further substantiate our hypothesis that the defects observed in PI3K-CA expressing nephrocytes are due to ectopic activation of Akt/mTOR signaling upon increased levels of PI(3,4,5)P3, we knocked down Akt or *Drosophila* Tor (dTOR) in PI3K-CA expressing nephrocytes. As depicted in Fig. 3F, G, J and Supplementary Fig. 3C, downregulation of Akt or dTOR rescued to a large extent the slit diaphragm defects as well as size differences in PI3K-CA expressing nephrocytes, further supporting the hypothesis that the dominant-negative function of PI(3,4,5)P3 is mediated by the Akt/mTOR pathway. We confirmed that this rescue effect was not due to an inhibition of biosynthesis of the PI3K-CA transgene, because downregulation of dTOR in larvae did not affect the expression of a GFP transgene but abolishes Akt phosphorylation (Supplementary Fig. 3K).

### Changes in PI(4,5)P2 but not in PI(3,4,5)P3 levels cause rapid defects

**Fig. 4:**
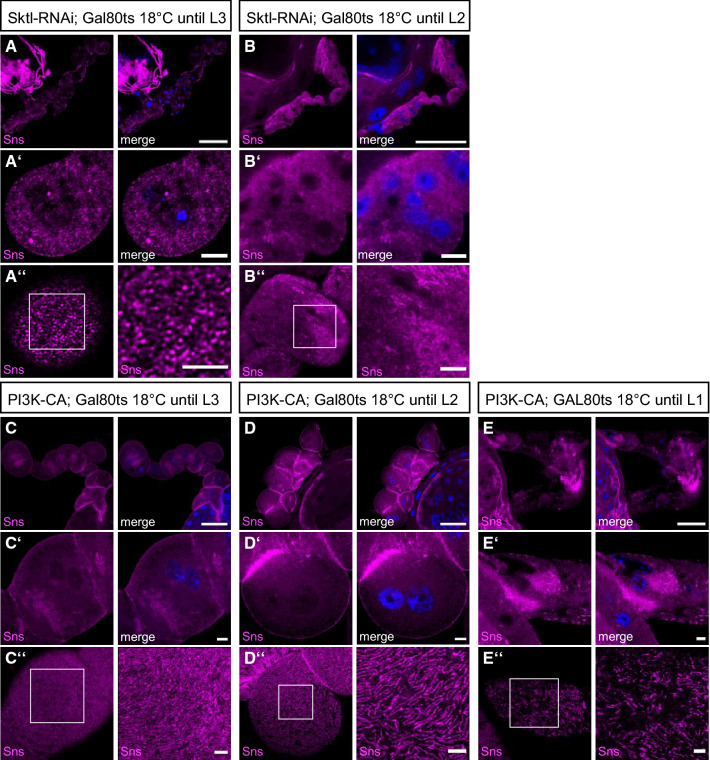
Rapid effects of PI(4,5)P2 reduction and PI(3,4,5)P3 accumulation. **A**–**E** Immunostainings of nephrocytes from 3rd instar larvae expressing GAL80ts together with sns::GAL4 and Sktl-RNAi (**A**, **B**) or PI3K-CA (**C**–**E**), which were first raised at 18 °C in order to suppress expression of the transgenes. After first (L1, **E**) or second (L2, **B** and **D**) instar larval stage, larvae were shifted to 29 °C prior to dissection to induce expression of the transgenes. In A and C, larvae were not shifted and expression of the transgene was suppressed by GAL80ts. Scale bars are 50 µm in **A**–**E**, 5 µm in **A**’-**E**’ and 2.5 µm in insets in **A**”–**E**”

To elucidate whether slit diaphragm defects are established early in development during formation of nephrocytes or whether PI(4,5)P2 and PI(3,4,5)P3 levels are also essential for the turnover and maintenance of slit diaphragms, we used a temperature-sensitive GAL80 (GAL80ts), which suppresses GAL4 activity at the permissive temperature at 18 °C. Raised at 18 °C, Sktl-RNAi suppressing nephrocytes exhibit moderate defects in surface Sns (accounting for some leakiness in the GAL80ts system), but no fusion phenotypes. Suppression of PI3K-CA by GAL80ts at 18 °C produced no phenotypes (Fig. 4A, C). After molting to L3, larvae were shifted to 29 °C for 24 h prior to dissection, inactivating the GAL80 and thus releasing GAL4, which induces the UAS transgene. In Sktl-RNAi expressing nephrocytes dissected from animals raised under these conditions, we observed similar defects in morphology/fusion as well as impaired Sns strands (Fig. 4B), indicating that PI(4,5)P2 is essential for the turnover/maintenance after the initial establishment of slit diaphragms during the development of nephrocytes. In contrast, short-term induction of PI3K-CA did not produce phenotypes comparable to continuous expression of this transgene but only mild reduction in slit diaphragms (Fig. 4D). Raising PI3K-CA expressing L2 larvae to 29 °C, thus allowing expression of PI3K-CA for 48 h, produced an intermediate phenotype with fusion defects and stronger slit diaphragm defects (Fig. 4E). These data indicate that the Akt/mTOR-mediated effect of ectopic PI(3,4,5)P3 production takes a longer time to get established, presumably due to the delay upon transcriptional reprogramming of the cell as a consequence of mTOR target activation.

## Discussion

Our findings demonstrate that PI(4,5)P2, but not PI(3,4,5)P3 is essential for nephrocyte function and slit diaphragm formation. Of note, PI(4,5)P2 is not evenly distributed in the entire plasma membrane but displays a strand-like pattern, partly colocalizing with Sns as a maker for slit diaphragms. Although PI(4,5)P2 has been found in other cell types at the entire plasma membrane—or, in epithelial cells, enriched in the apical plasma membrane domain—there is increasing evidence that this phospholipid is concentrated in distinct microdomains of the plasma membrane [discussed by 52, 53]: in cultured fibroblasts, freeze-fracture membrane preparation and subsequent electron microscopy revealed three distinct pools of PH(PLCδ) at the rim of caveolae, in coated pits and at the free plasma membrane [54]. Notably, these three pools exhibited different kinetics upon regulatory stimuli, suggesting different types of regulation. PI(4,5)P2 was also reported to accumulate in lipid rafts of distinct (phospho)lipid and cholesterol composition within the plasma membrane, promoting local actin remodeling or receptor clustering [55–57]. Sarmento et al. observed a Ca(2+)-dependent PI(4,5)P2 clustering in liposomes in vitro under physiological Ca(2+) and PI(4,5)P2 concentrations [58]. Thus, PI(4,5)P2 may accumulate in distinct microdomains of the plasma membrane adjacent to slit diaphragms to regulate vesicle trafficking—to the plasma membrane by inducing fusion of vesicles and from the plasma membrane by regulating endocytosis. The dramatic phenotypes observed in Sktl-RNAi expressing nephrocytes underline the critical role of PI(4,5)P2 as an important regulator of these processes. Notably, the human homologue of Sktl, PIP5Kα, was described to be recruited by the Chloride Intracellular Channel 5 (CLIC5A) to cortical Ezrin, inducing clusters of PI(4,5)P2 in the plasma membrane of COS-7 cells [59]. In podocytes, Ezrin is part of the Ezrin-NHERF2-Podocalyxin complex, an essential component of the glycocalyx. Furthermore, in glomeruli of CLIC5A-deficient mice, cortical Ezrin/NHERF2 as well as glomerular Podocalyxin are reduced [59]. Another hint to an important role of PI(4,5)P2 in regulating podocyte morphology comes from a study reporting that the PI5P-Phosphatase Ship2 can be recruited and activated by Nephrin via Nck-Pak1-Filamin in cultured human podocytes [60]. Ship2 dephosphorylates PI(3,4,5)P3 to PI(3,4)P2, thus its activation by Nephrin in this systems results in an increase of PI(3,4)P2, which activates Lamellipodin, a regulator of Ena/Vasp proteins, resulting in the formation of lamellipodia. Finally, the Nephrin/Ship2 interaction was increased in a podocyte injury model in vivo, suggesting that lamellipodia formation upon Nephrin-mediated Ship2 activation contributes to foot process effacement observed upon podocyte damage. However, it remains unclear how the Ship2-regulated balance between PI(3,4,5)P3 and PI(3,4)P2 at the Nephrin-complex contributes to slit diaphragm assembly/maintenance and podocyte function under physiological conditions.

PI(4,5)P2 as well as PI(3,4,5)P3 are capable of regulating the actin cytoskeleton by recruiting and activating the small GTPases Rac1 and Cdc42 as well as proteins of the WASP family [21–23]. Notably, a coordinated actin cytoskeleton remodeling is essential for cortical Nephrin localization and slit diaphragm assembly in *Drosophila* nephrocytes [61, 62] as well as in mammalian podocytes [63]. Vice versa, activated Nephrin recruits PI3K resulting in Rac1 activation, actin branching and lamellipodia formation in cultured rat podocytes [25]. PTEN is downregulated in podocytes of patients suffering from diabetic nephropathy and inhibition or podocyte-specific knockout of PTEN in mice results in cytoskeleton rearrangements, foot process effacement and proteinuria [64].

Apart from their impact on the actin cytoskeleton, PI(4,5)P2 and PI(3,4,5)P3-activated Rac1/Cdc42 and actin regulators are essential for remodeling and stability of tight junctions as well as adherens junctions in classical epithelia [reviewed by 65]. Increasing evidence suggests that the slit diaphragms connecting the foot processes of neighboring podocytes emerge from transformation of the tight junctions of the epithelial podocyte progenitor cells [66]. Indeed, several proteins of the adherens and tight junctions can also be found to be components of the slit diaphragm, e.g. ZO-1, Crumbs, PAR/aPKC complex [1, 8, 10, 11, 67–73]. Therefore, it is likely that changes in PI(4,5)P2 and PI(3,4,5)P3 affect slit diaphragm formation and maintenance/stability like they affect adherens junctions/tight junctions in classical epithelia. Our data suggest that nephrocytes exhibit a junctional (slit diaphragm) versus non-junctional polarity regarding the phospholipid composition of the plasma membrane and the localization of polarity regulators as shown before [as shown before in 9].

### Supplementary Information

Below is the link to the electronic supplementary material.

Supplementary file1 Biosensors for PI(4,5)P2 and PI(3,4,5)P3 display distinct localization patterns. Related to Fig. 1. **A**, **B** Garland nephrocytes expressing PH(Akt)-GFP were co-stained with Talin, showing a substantial colocalization. **C** Quantification of Sns strands of nephrocytes expressing PH(PLCδ)-mCherry or PH(Akt)-GFP demonstrates no defects in slit diaphragms. 5 lines/nephrocyte and at least 5 nephrocytes were quantified per genotype. Significance was determined by Kruskal-Wallis test and Dunn’s correction: n.s. not significant. **D** Quantification of colocalization of the indicated transgenes with Baz and Sns (related to Fig. 1B). **E**, **F** Simultaneous expression of PH(PLCδ)-mCherry and PH(Akt)-GFP reveal limited overlap. Scale bars are 5 µm and 1 µm in insets (PDF 598 KB)

Supplementary file2 Overexpression of Sktl does not affect slit diaphragm assembly. Related to Fig. 2. **A** Nephrocytes expressing PH(PLCδ)-mCherry and Sktl-RNAi were stained with Baz and Sns. **B**, **C** Control (**B**) or Sktl RNAi-expressing nephrocytes (**C**) were stained with Rab5 (marker for early endosomes), Rab7 (labelling late endosomes and lysosomes) and Rab11 (marker for recycling endosomes). **D**, **E** Western Blot and quantification of ANP-2xGFP in lysates of whole L3 larvae expressing control RNAi, Sktl RNAi or PI3K-CA. Significance was determined by repeated measures ANOVA with Bonferroni’s multiple comparison test: n.s. not significant. **F** Endocytosis assays with FITC-Albumin. Nephrocytes were pulsed with FITC-Albumin for 15 min and subsequently chased for 2 h. At least 120 nephrocytes from at least 25 independent larvae were quantified. Significance was determined by Kruskal-Wallis test and Dunn’s correction: ***p<0.001. **G** Immunostaining of Skittles-Myc, PH(PLCδ)-mCherry and Sns in nephrocytes. **H** Nephrocytes overexpressing Sktl were stained with the indicated antibodies. **I** Overexpression of Sktl results in increased accumulation of PH(PLCδ)-mCherry at the plasma membrane. **J** Downregulation of Sec3 results in disturbed Sns- and Talin strands. Scale bars are 5 µm in **A**–**J** except of H, 25 µm in **H** and 2,5 µm in B’’, C’’ and insets in G’’, J and J’. Error bars are standard error of the means (PDF 265 KB)

Supplementary file3 Decrease of PI(3,4,5)P3 does not affect slit diaphragms. Related to Fig. 3. **A**, **B** Nephrocytes overexpressing a dominant negative PI3K (PI3K-DN, **A**) or PTEN (**B**) were stained with the indicated antibodies. **C** RNAi targeting dTOR was expressed in nephrocytes together with expression of PI3K-CA. **D** PH(Akt)-GFP in nephrocytes expressing PI3K-CA display an enhanced cortical localization. **E**–**G** Control (**E**) and PI3K-CA expressing (**F**) nephrocytes were stained with Sns and p-S6K. p-S6K intensity per nephrocyte was quantified by background substracted mean grey value x area (**G**). Significance was determined by Mann Whitney test: ***p < 0.001. **H** To determine fusion events, the number of nuclei per nephrocyte was quantified in control and PI3K-CA expressing nephrocytes. Significance was determined by Wilcoxon signed rank test: *** *p* < 0.001. **I** Expression of PI3K-CA with dot::GAL4 results in displacement of Baz and Talin from the membrane and reduction of Sns strands at the surface. **J** The number of nephrocytes per larvae was quantified. For each genotype, at least ten independent larvae were quantified. **K** Western blot of larvae expressing GFP together with control RNAi or RNAi against dTOR. Scale bars are 25 µm in A, B, C and I and 5 µm in A’, A’’, B’, B’’, C’, C’’, D, E, I’, I’’ and 2,5 µm in inset in I’’. Error bars are standard error of the means (PDF 579 KB)

## Data Availability

All data are available in main und supplemental figures.
