# Supplementary figures and images for: PI(4,5)P2 controls slit diaphragm formation and endocytosis in Drosophila nephrocytes
Source: Cell Mol Life Sci. 2022 Apr 18;79(5):248. doi: 10.1007/s00018-022-04273-7 (PMC9016003; doi:10.1007/s00018-022-04273-7)

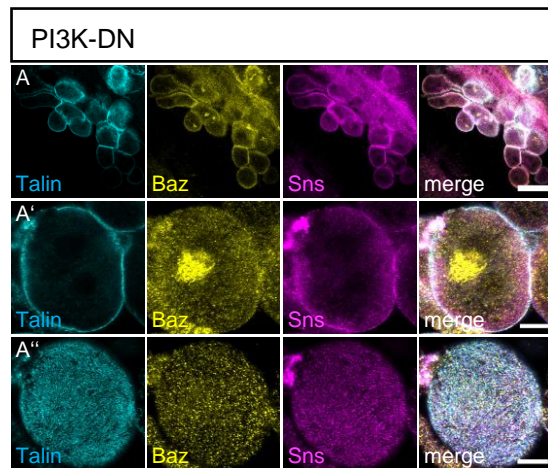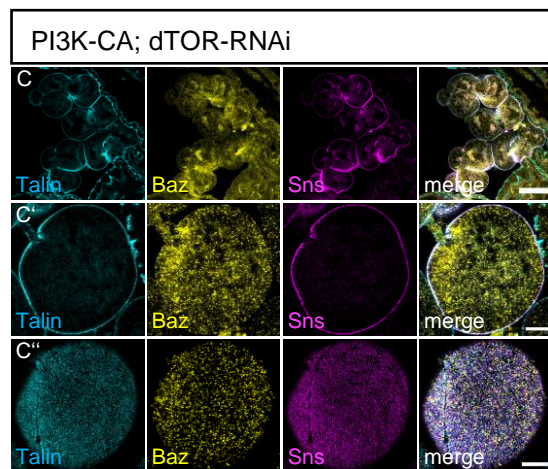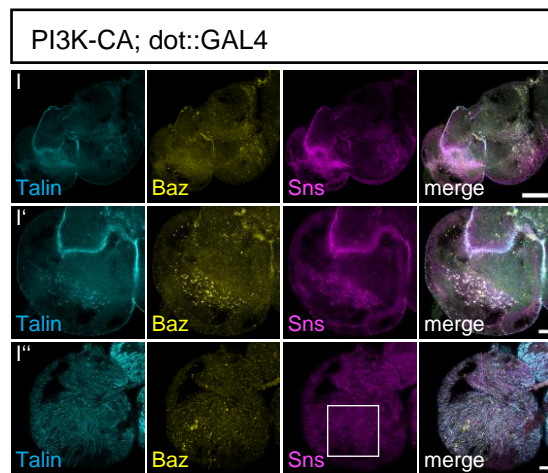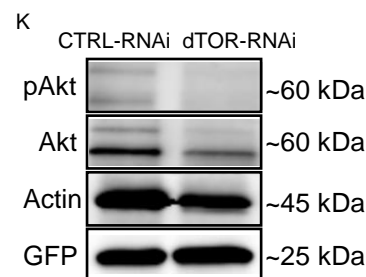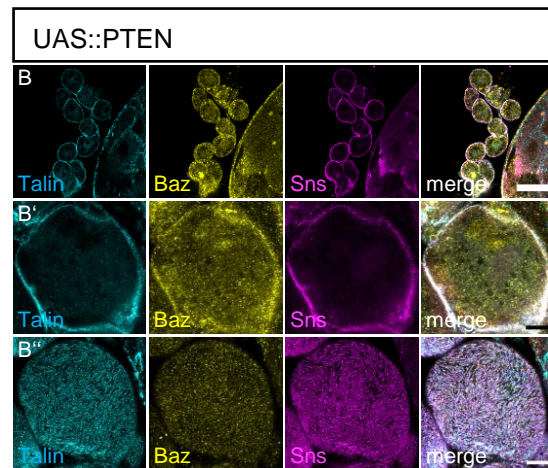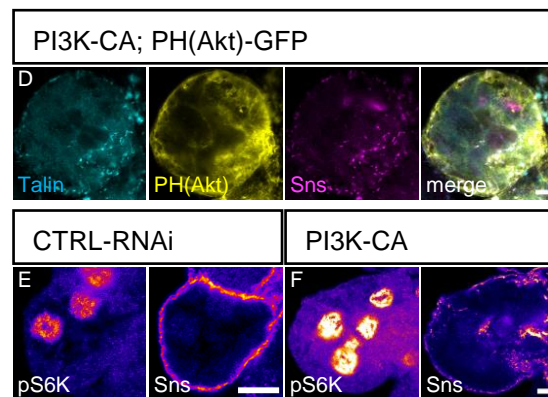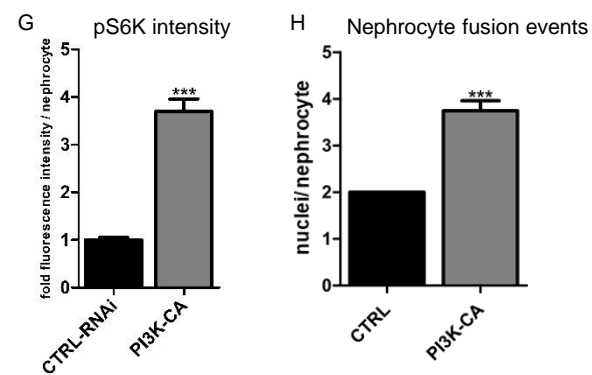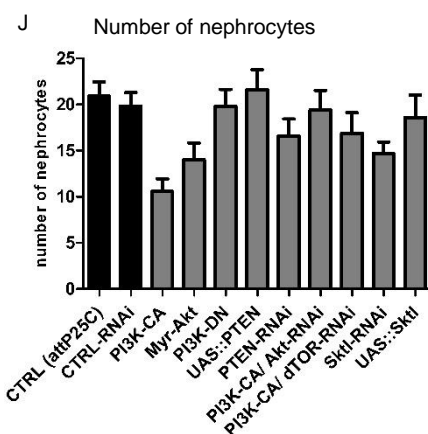

Supplement: Supplementary file 1 — Supplementary file1 Biosensors for PI(4,5)P2 and PI(3,4,5)P3 display distinct localization patterns. Related to Fig. 1. A, B Garland nephrocytes expressing PH(Akt)-GFP were co-stained with Talin, showing a substantial colocalization. C Quantification of Sns strands of nephrocytes expressing PH(PLCδ)-mCherry or PH(Akt)-GFP demonstrates no defects in slit diaphragms. 5 lines/nephrocyte and at least 5 nephrocytes were quantified per genotype. Significance was determined by Kruskal-Wallis test and Dunn’s correction: n.s. not significant. D Quantification of colocalization of the indicated transgenes with Baz and Sns (related to Fig. 1B). E, F Simultaneous expression of PH(PLCδ)-mCherry and PH(Akt)-GFP reveal limited overlap. Scale bars are 5 µm and 1 µm in insets (PDF 598 KB) [file 18_2022_4273_MOESM1_ESM.pdf]

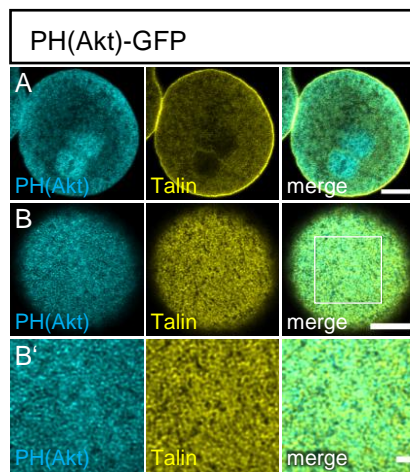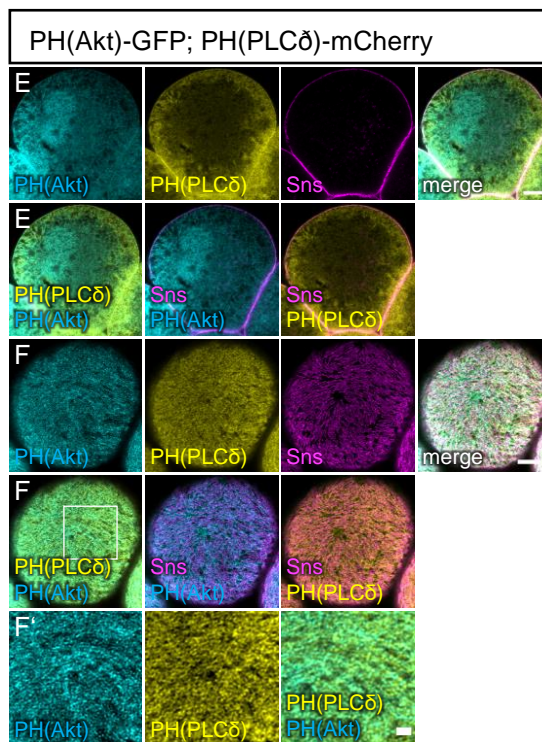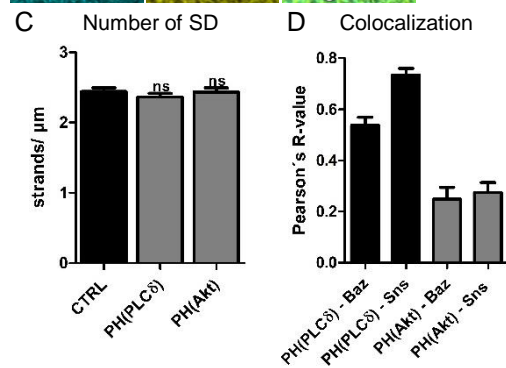

Supplement: Supplementary file 2 — Supplementary file2 Overexpression of Sktl does not affect slit diaphragm assembly. Related to Fig. 2. A Nephrocytes expressing PH(PLCδ)-mCherry and Sktl-RNAi were stained with Baz and Sns. B, C Control (B) or Sktl RNAi-expressing nephrocytes (C) were stained with Rab5 (marker for early endosomes), Rab7 (labelling late endosomes and lysosomes) and Rab11 (marker for recycling endosomes). D, E Western Blot and quantification of ANP-2xGFP in lysates of whole L3 larvae expressing control RNAi, Sktl RNAi or PI3K-CA. Significance was determined by repeated measures ANOVA with Bonferroni’s multiple comparison test: n.s. not significant. F Endocytosis assays with FITC-Albumin. Nephrocytes were pulsed with FITC-Albumin for 15 min and subsequently chased for 2 h. At least 120 nephrocytes from at least 25 independent larvae were quantified. Significance was determined by Kruskal-Wallis test and Dunn’s correction: ***p<0.001. G Immunostaining of Skittles-Myc, PH(PLCδ)-mCherry and Sns in nephrocytes. H Nephrocytes overexpressing Sktl were stained with the indicated antibodies. I Overexpression of Sktl results in increased accumulation of PH(PLCδ)-mCherry at the plasma membrane. J Downregulation of Sec3 results in disturbed Sns- and Talin strands. Scale bars are 5 µm in A–J except of H, 25 µm in H and 2,5 µm in B’’, C’’ and insets in G’’, J and J’. Error bars are standard error of the means (PDF 265 KB) [file 18_2022_4273_MOESM2_ESM.pdf]
