# Supplementary material for: PI(4,5)P2 controls slit diaphragm formation and endocytosis in Drosophila nephrocytes
Source: Cell Mol Life Sci. 2022 Apr 18;79(5):248. doi: 10.1007/s00018-022-04273-7 (PMC9016003; doi:10.1007/s00018-022-04273-7)
Supplement: Supplementary file 3 — Supplementary file3 Decrease of PI(3,4,5)P3 does not affect slit diaphragms. Related to Fig. 3. A, B Nephrocytes overexpressing a dominant negative PI3K (PI3K-DN, A) or PTEN (B) were stained with the indicated antibodies. C RNAi targeting dTOR was expressed in nephrocytes together with expression of PI3K-CA. D PH(Akt)-GFP in nephrocytes expressing PI3K-CA display an enhanced cortical localization. E–G Control (E) and PI3K-CA expressing (F) nephrocytes were stained with Sns and p-S6K. p-S6K intensity per nephrocyte was quantified by background substracted mean grey value x area (G). Significance was determined by Mann Whitney test: ***p < 0.001. H To determine fusion events, the number of nuclei per nephrocyte was quantified in control and PI3K-CA expressing nephrocytes. Significance was determined by Wilcoxon signed rank test: *** p < 0.001. I Expression of PI3K-CA with dot::GAL4 results in displacement of Baz and Talin from the membrane and reduction of Sns strands at the surface. J The number of nephrocytes per larvae was quantified. For each genotype, at least ten independent larvae were quantified. K Western blot of larvae expressing GFP together with control RNAi or RNAi against dTOR. Scale bars are 25 µm in A, B, C and I and 5 µm in A’, A’’, B’, B’’, C’, C’’, D, E, I’, I’’ and 2,5 µm in inset in I’’. Error bars are standard error of the means (PDF 579 KB) [file 18_2022_4273_MOESM3_ESM.pdf]

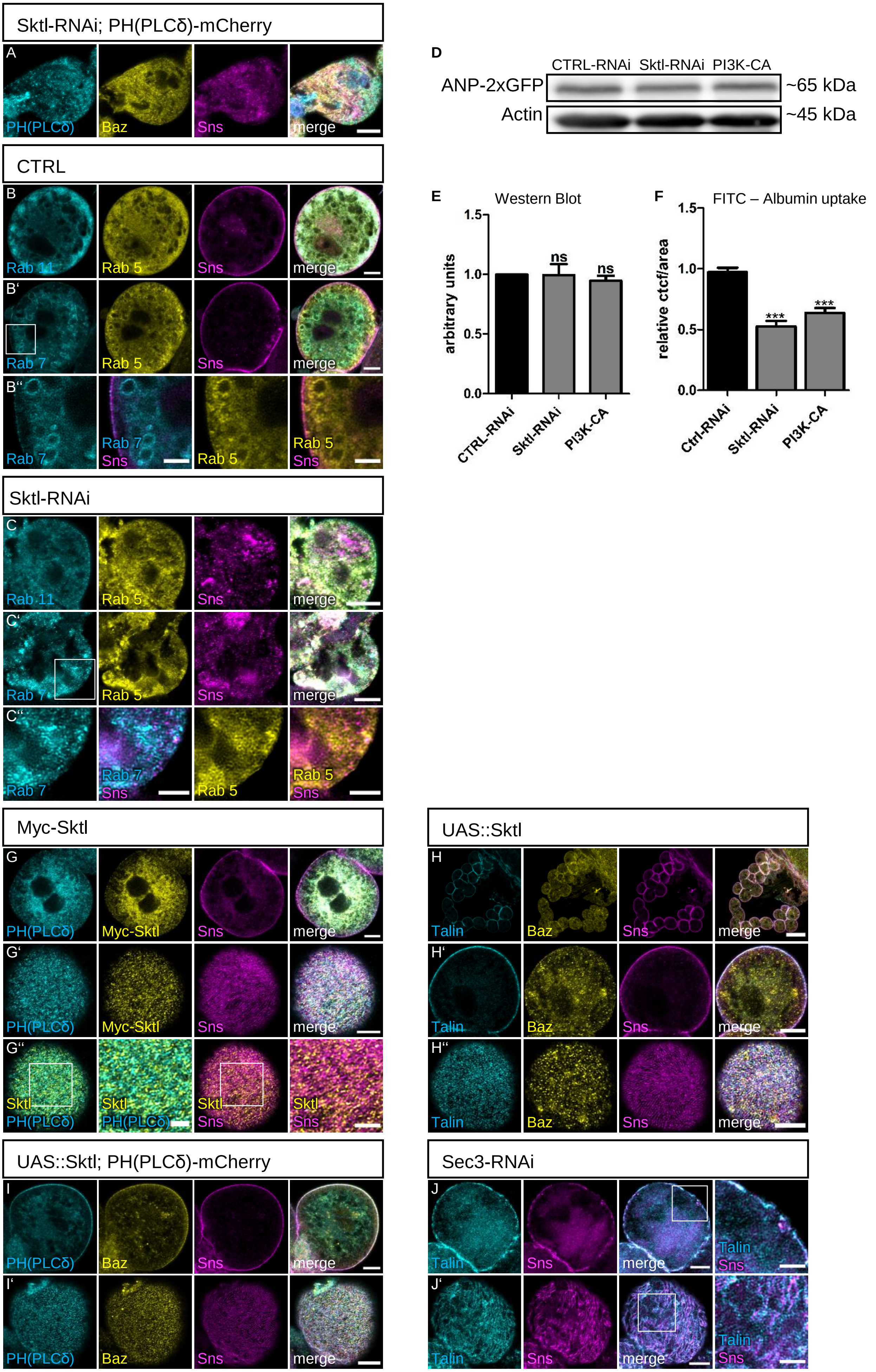

Sktl-RNAi; PH(PLCδ)-mCherry
A
PH(PLCδ)
Baz
Sns
merge
CTRL
B
Rab 11
Rab 5
Sns
merge
B‘
Rab 7
Rab 5
Sns
merge
B“
Rab 7
Rab 7
Sns
Rab 5
Rab 5
Sns
Sktl-RNAi
C
Rab 11
Rab 5
Sns
merge
C‘
Rab 7
Rab 5
Sns
merge
C“
Rab 7
Rab 7
Sns
Rab 5
Rab 5
Sns
D
CTRL-RNAi
Sktl-RNAi
PI3K-CA
ANP-2xGFP
~65 kDa
Actin
~45 kDa
E
Western Blot
arbitrary units
1.5
1.0
0.5
0.0
ns
ns
CTRL-RNAi
Sktl-RNAi
PI3K-CA
F
FITC – Albumin uptake
relative ctcf/area
1.5
1.0
0.5
0.0
***
***
Ctrl-RNAi
Sktl-RNAi
PI3K-CA
Myc-Sktl
G
PH(PLCδ)
Myc-Sktl
Sns
merge
G‘
PH(PLCδ)
Myc-Sktl
Sns
merge
G“
Sktl
PH(PLCδ)
Sktl
PH(PLCδ)
Sktl
Sns
Sktl
Sns
UAS::Sktl
H
Talin
Baz
Sns
merge
H‘
Talin
Baz
Sns
merge
H“
Talin
Baz
Sns
merge
UAS::Sktl; PH(PLCδ)-mCherry
I
PH(PLCδ)
Baz
Sns
merge
I‘
PH(PLCδ)
Baz
Sns
merge
Sec3-RNAi
J
Talin
Sns
merge
Talin
Sns
J‘
Talin
Sns
merge
Talin
Sns
